# Perioperative Short‐Term Glucocorticoids Do Not Increase Incidence of Complications after Total Joint Arthroplasty in Patients with Rheumatoid Arthritis

**DOI:** 10.1111/os.14150

**Published:** 2024-07-01

**Authors:** Yahao Lai, Chao Huang, Yongrui Cai, Zichuan Ding, Jiaxuan Fan, Zeyu Luo, Zongke Zhou

**Affiliations:** ^1^ Department of Orthopaedic Surgery West China Hospital of Sichuan University Chengdu China

**Keywords:** arthroplasty, complication, glucocorticoids, opioids, pain, rheumatoid arthritis

## Abstract

**Objectives:**

The safety and analgesic efficacy of perioperative glucocorticoids have been established for patients without rheumatoid arthritis. Therefore, our study aims to investigate whether similar benefits can be observed in patients with rheumatoid arthritis undergoing total joint arthroplasty. Specifically, we aim to explore the impact of perioperative glucocorticoid use on postoperative complications, opioid consumption, incidence of hypotension, hyperglycemia, 30‐day mortality, and 90‐day re‐admission in this patient population.

**Methods:**

Approval for the study protocol was obtained from the Medical Research Ethics Committee at Sichuan University, aligning with the principles outlined in the Declaration of Helsinki. We retrospectively analyzed a consecutive series of patients with rheumatoid arthritis who underwent total joint arthroplasty at our medical center between November 2009 and April 2021 and who were not on chronic glucocorticoid therapy before surgery. Those who received glucocorticoids at any time during hospitalization were compared to those who did not in terms of acute complications within 90 days after surgery as well as postoperative rescue opioid consumption, hypotension, and hyperglycemia during hospitalization. The two groups were also compared in terms of overall duration of hospitalization, all‐cause mortality within 30 days, and readmission for any reason within 90 days. Continuous data were assessed for significance using the independent‐samples t test. Categorical data were assessed using the Pearson chi‐squared test.

**Results:**

Of the 849 patients included in the analysis, 598 administered perioperative glucocorticoids and 251 did not. Prior to surgery, the two groups did not differ significantly in any clinicodemographic variable that we examined. The incidence of acute postoperative complications (2.3% vs. 4.0%, *p* = 0.187) and acute postoperative infection (2.0% vs. 2.8%, *p* = 0.482) was comparable between those who received perioperative glucocorticoids and those who did not, but the former group exhibited a significantly lower incidence of rescue opioid use (17.9% vs. 44.6%, *p* < 0.001) as well as significantly lower total rescue opioid consumption (4.7 ± 2.1 mg vs. 8.9 ± 4.6 mg, *p* < 0.001). However, the two groups showed similar incidences of postoperative hypotension, hyperglycemia, 30‐day mortality, and 90‐day re‐admission.

**Conclusion:**

Perioperative glucocorticoids may reduce the need for rescue opioids after total joint arthroplasty of rheumatoid arthritis patients, without increasing the incidence of acute complications, hypotension or hyperglycemia.

## Graphical abstract

Perioperative use of glucocorticoids in arthroplasty has many benefits, but its use in patients with rheumatoid arthritis remains controversial. Our study confirms that perioperative use of glucocorticoids can reduce the need for rescue opioids after total joint arthroplasty in patients with rheumatoid arthritis without increasing the incidence of acute complications, hypotension, and hyperglycemia.

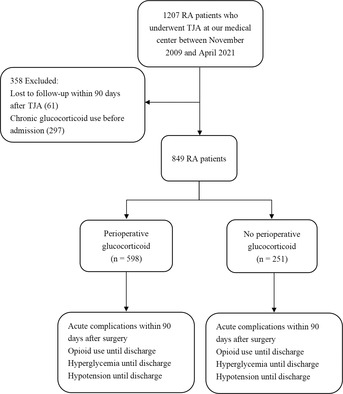

## Introduction

Total joint arthroplasty represents a highly efficacious intervention for end‐stage hip or knee disease, and perioperative corticosteroids, as part of contemporary multimodal treatment regimens, are often administered in order to relieve postoperative pain, reduce the risk of postoperative complications, and enhance recovery.^1^ Numerous randomized controlled trials^2^, ^3^ and meta‐analyses^4^, ^5^, ^6^, ^7^ have consistently demonstrated that perioperative glucocorticoids can significantly reduce postoperative pain, opioid use, nausea, and vomiting without increasing risk of postoperative complications. However, most of these studies have focused on patients with osteoarthritis of hip or knee other than rheumatoid arthritis. As a result, the safety and efficacy of perioperative glucocorticoids remain controversial for patients with rheumatoid arthritis undergoing total joint arthroplasty.

The necessity of addressing this question arises from the fact that while the latest guidelines from the American College of Rheumatology recommend the continuation of chronic preoperative glucocorticoid doses throughout the perioperative period,^8^ such perioperative use has been associated with a higher risk of acute complications such as infection after total joint arthroplasty in patients with rheumatoid arthritis.^9^, ^10^, ^11^ Furthermore, there is a lack of studies on the safety and efficacy of perioperative glucocorticoids for patients with rheumatoid arthritis who were not previously on chronic glucocorticoid therapy before arthroplasty.

Therefore, this study aims to compare patients who received glucocorticoids preoperatively in our center with those who did not in terms of (i) incidence of postoperative complications; (ii) risk and dose of rescue opioids needed; and (iii) incidence of hyperglycemia and hypotension.

## Methods and Materials

### Patients

With approval from the Ethics Committee for Biomedical Research at our hospital (approval 2023–2005), we retrospectively analyzed a consecutive series of patients who (i) were diagnosed with rheumatoid arthritis based on the criteria of the American College of Rheumatology and European League Against Rheumatism^12^; (ii) were at least 18 but no older than 80 years old; (iii) were not on chronic glucocorticoid therapy before admission; (iv) underwent primary, unilateral, total arthroplasty of the hip or knee at our hospital between November 2009 and April 2021. Patients were excluded if they (i) were lost to follow‐up within 90 days after surgery; (ii) required specialized joint prostheses due to having severe preoperative bone defects. The Ethics Committee waived the requirement for informed consent because, at the time of treatment, patients or their legal guardians consented to the analysis and publication of anonymized medical data for research purposes.

### Interventions and Follow‐Up

All arthroplasties in this study were conducted using standard procedures under the same analgesic protocol. All patients received antibiotics during the perioperative period to prevent infection. All patients were treated with the same perioperative strategies, including tranexamic acid, pain management, thrombosis prevention, and functional rehabilitation. Selective COX‐2 inhibitors have been used for pain relief. For thrombosis prophylaxis, we administered low‐molecular‐weight heparin after surgery until discharge. Subsequently, apixaban was continued for 2 weeks. Primary total joint arthroplasty was performed by five experienced joint surgeons using a standardized surgical approach to ensure similar surgical trauma among all patients. Nurses regularly assessed postoperative pain severity using a visual rating scale from 0 to 10. Patients indicating a score of 4 were provided with oral oxycodone, while those indicating a score of 6 or higher received intramuscular morphine until their pain rating fell below 4. Blood glucose and blood pressure levels were monitored daily during hospitalization. Patients with poorly controlled blood pressure and/or blood glucose at discharge were advised to self‐monitor regularly.

The outpatient clinic visits were scheduled for all patients at 1, 3, 6, and 12 months post‐discharge, followed by annual check‐ups. During each follow‐up appointment, comprehensive physical examinations and X‐ray assessments were conducted to detect any potential postoperative complications.

### Data Collection and Outcomes

The data collected upon admission encompassed a range of clinicodemographic characteristics, including disease‐modifying anti‐rheumatic drugs, Charlson comorbidity index, diabetes status, and type of anesthesia employed. Intraoperative blood loss and blood transfusion were quantified based on the anesthesia record sheet. Dosages of all glucocorticoids administered during hospitalization were standardized to dexamethasone equivalents. Patients who received any glucocorticoids during hospitalization were compared to those who received none in terms of the primary outcome, which was defined as acute complications occurring within 90 days after surgery. These complications encompassed myocardial infarction, deep vein thrombosis, pulmonary embolism, pulmonary infection, sepsis, prosthetic joint infection, superficial surgical site infection, dislocation and periprosthetic fracture. The two groups were compared in terms of the following postoperative secondary outcomes until discharge: cumulative postoperative opioid dose; incidence of hyperglycemia, defined as blood glucose ≥180 mg/ld.; and incidence of postoperative hypotension. The two groups were also compared in terms of overall duration of hospitalization, all‐cause mortality within 30 days, and readmission for any reason within 90 days.

### Statistical Analysis

Continuous data were reported as mean ± standard deviation (SD), or median (interquartile range), and intergroup differences were assessed for significance using the independent‐samples *t* test. Categorical data were reported as *n* (%), and intergroup differences were assessed using the Pearson chi‐squared test. All statistical analyses were performed using SPSS 26.0 (IBM, Armonk, NY, USA), and results associated with *P* < 0.05 were considered significant.

## Results

**Figure 1:**
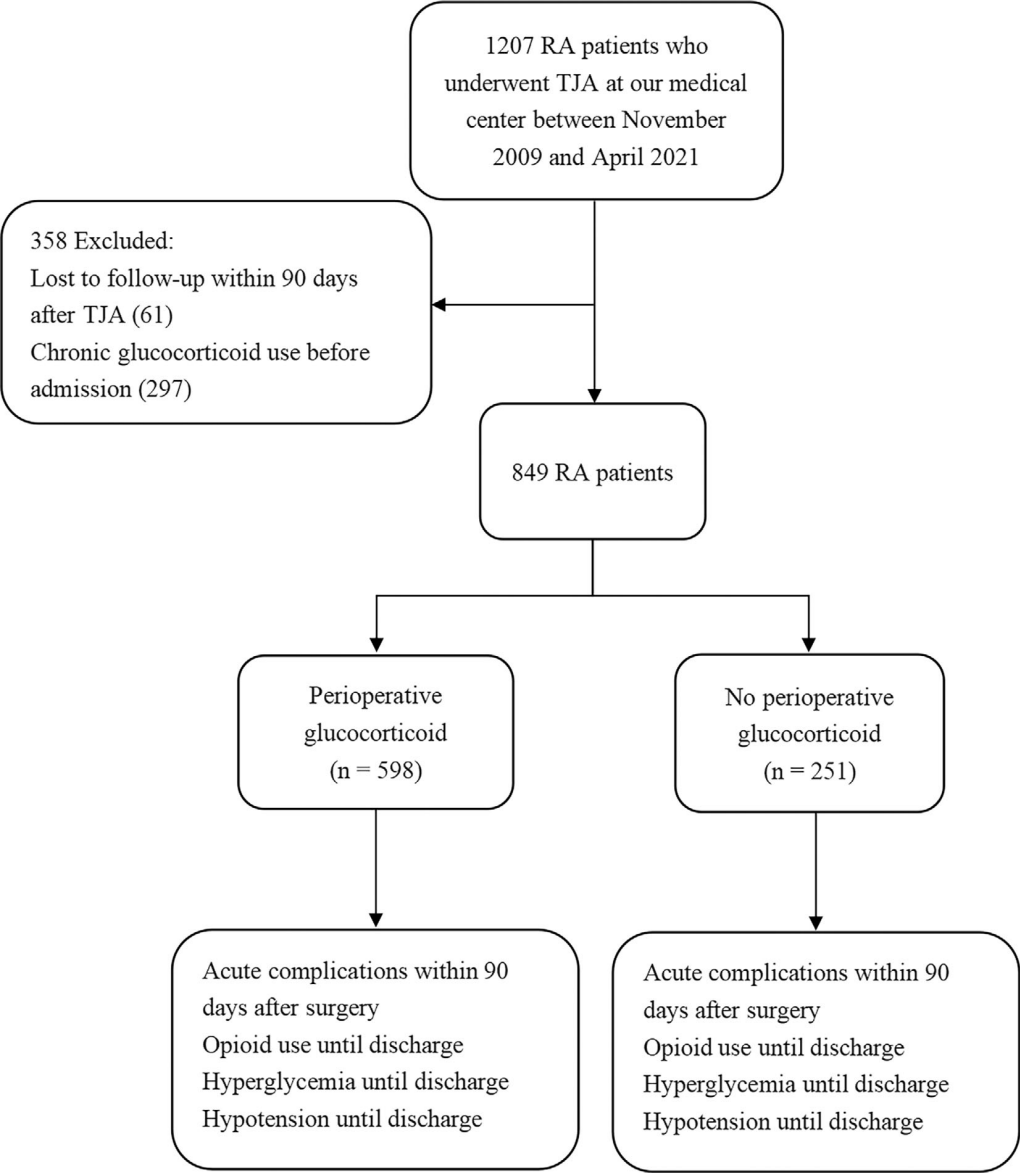
Flow diagram of patient selection and analysis. RA, rheumatoid arthritis; TJA, total joint arthroplasty.

Of 1207 rheumatoid arthritis patients whom we screened for enrollment, we excluded 297 because of chronic glucocorticoid use before admission and another 61 because they were lost to follow‐up (Figure 1). In the end, we analyzed 849 patients, of whom 598 (70.4%) received perioperative glucocorticoids and 251 (29.6%) did not. The two groups did not exhibit statistically significant differences in terms of sex, age, body mass index, Charlson comorbidity index, or prevalence of diabetes (Table 1). In addition, there were no statistically significant differences in intraoperative conditions including intraoperative blood loss and blood transfusion between the two groups. Among those who received perioperative glucocorticoids, most received dexamethasone (73.6%), followed by methylprednisolone (16.2%) and hydrocortisone (10.2%).

**Table 1:** Clinicodemographic characteristics of patients, stratified by perioperative glucocorticoid use.

Characteristic	Perioperative glucocorticoids		*p*
	Yes (*n* = 598)	No (*n* = 251)	
Sex			0.071
Male	87 (14.5)	49 (19.5)	
Female	511 (85.5)	202 (80.5)	
Age, years	54.5 ± 11.7	55.3 ± 12.0	0.523
Body mass index, kg/m^2^	22.6 ± 3.6	22.0 ± 3.9	0.111
Charlson comorbidity index			0.607
< 2	264 (44.1)	106 (42.2)	
≥ 2	334 (55.9)	145 (57.8)	
American Society of Anesthesiologists grade			0.303
< 3	490 (81.9)	213 (84.9)	
≥ 3	108 (18.1)	38 (15.1)	
Medication history			0.407
DMARDs	107 (17.9)	39 (15.5)	
Other drugs	491 (82.1)	212 (84.5)	
Diabetes			0.078
No	543 (90.8)	237 (94.4)	
Yes	55 (9.2)	14 (5.6)	
Intraoperative conditions			
Intraoperative blood loss (mL)	121.3 ± 52.4	118.9 ± 47.9	0.533
No. patients requiring blood transfusion	46 (7.7)	21 (8.4)	0.740

*Note*: Values are *n* (%) or mean ± SD, unless otherwise noted.

Abbreviation: DMARD, disease‐modifying anti‐rheumatic drug.

Patients who received perioperative glucocorticoids showed a similar incidence of acute complications within 90 days after surgery as those who did not receive glucocorticoids (2.3 vs. 4.0%, *p* = 0.187), and the incidence of specific complications did not differ significantly between the two groups (Table 2). The incidence of overall acute postoperative infection (2.0% vs. 2.8%, *p* = 0.482), periprosthetic joint infection (0.8% vs. 0.8%, *p* = 1.000), and superficial infection (1.2% vs. 2.0%, *p* = 0.335) were similar between the two groups. The two groups were also similar to each other in rates of mortality within 30 days and of readmission within 90 days.

**Table 2:** Comparison of acute complications within 90 days after total joint arthroplasty between patients who received perioperative glucocorticoids or not.

Complication	Perioperative glucocorticoids		*p*
	Yes (*n* = 598)	No (*n* = 251)	
All complications combined	14 (2.3)	10 (4.0)	0.187
Deep vein thrombosis	1 (0.2)	0	1.000
Pulmonary embolism	0	0	ND
Infection	12 (2.0)	7 (2.8)	0.482
Periprosthetic joint infection	5 (0.8)	2 (0.8)	1.000
Superficial infection	7 (1.2)	5 (2.0)	0.355
Periprosthetic fracture	0	1 (0.4)	0.296
Aseptic loosening	0	0	ND
30‐day mortality	0	0	ND
90‐day readmission	8 (1.3)	6 (2.4)	0.272

*Note*: Values are *n* or *n* (%), unless otherwise noted.

Abbreviation: ND, not done.

A significantly larger proportion of patients who did not receive perioperative glucocorticoids required rescue opioids (44.6 vs. 17.9%, *p* < 0.001), and per‐patient opioid consumption was significantly higher among those who did not receive glucocorticoids (8.9 ± 4.6 vs. 4.7 ± 2.1 mg, *p* < 0.001; Table 3). The two groups did not differ significantly in incidence of postoperative hyperglycemia or hypotension during hospitalization.

**Table 3:** Comparison of opioid consumption, hyperglycemia and hypotension after total joint arthroplasty between patients who received perioperative glucocorticoids or not.

Complications	Perioperative glucocorticoids		*p*
	Yes (*n* = 598)	No (*n* = 251)	
No. patients requiring rescue opioids	107 (17.9)	112 (44.6)	< 0.001
Mean rescue opioid dose, mg *	4.7 ± 2.1	8.9 ± 4.6	< 0.001
Hyperglycemia	315 (52.7)	137 (54.6)	0.661
Hypotension	2 (0.3)	3 (1.2)	0.135

*Note*: Values are n (%) or mean ± SD, unless otherwise noted.

* Morphine equivalents.

## Discussion

Our retrospective analysis of a reasonably large population of patients with rheumatoid arthritis undergoing total joint arthroplasty suggests perioperative glucocorticoids use in these individuals did not increase incidence of acute complications, hypotension, and hyperglycemia after surgery, but reduce the postoperative need for rescue opioids.

### Postoperative Complications

Our findings align with numerous studies conducted on patients without rheumatoid arthritis who underwent total joint arthroplasty, indicating that the perioperative use of glucocorticoids did not significantly impact the occurrence of postoperative complications.^4^, ^13^, ^14^, ^15^, ^16^ Our study further contributed valuable evidence to support the perioperative use of glucocorticoids in patients with rheumatoid arthritis. However, our results differ from a single study that associated higher perioperative glucocorticoid dosage with an increased risk of postoperative complications.^11^ We compared the differences in the cohorts included in our study and this study, and it is worth noting that this particular study included patients who were already receiving chronic glucocorticoid treatment prior to surgery, which may have contributed to the observed heightened risk.^10^

### Consumption of Opioids

The administration of perioperative glucocorticoids in patients with rheumatoid arthritis resulted in a decreased requirement for rescue opioids. Several studies have reported that in patients without rheumatoid arthritis, perioperative glucocorticoids can reduce the need for opioid rescue^17^ as well as dampen pain and inflammation.^18^ However, none of these studies included patients with rheumatoid arthritis. Given the potentially heightened inflammatory activity in patients with rheumatoid arthritis, our study contributes to the existing evidence supporting glucocorticoid treatment to relieve postoperative pain and reduce opioid consumption.

### Hyperglycemia and Hypotension

Perioperative glucocorticoids did not significantly increase the incidence of hyperglycemia in our patients following total joint arthroplasty. This finding contradicts previous studies that have linked glucocorticoid use to hyperglycemia after total joint arthroplasty in diabetic patients,^19^, ^20^ but not in patients with well‐controlled glycemic levels.^21^ Considering the presence of abnormal glucose metabolism in diabetic patients, the administration of corticosteroids at doses exceeding physiological levels may disrupt the delicate balance and result in an increased incidence of hyperglycemia. However, this issue may be ameliorated in non‐diabetic patients. Similarly, perioperative glucocorticoids did not significantly affect incidence of hypotension in our patients after total joint arthroplasty. While physiological doses of perioperative glucocorticoids appear not to alter risk of adrenal insufficiency after orthopaedic surgery,^22^ higher doses have been recommended as a way to prevent hypotension.^23^ Future studies should continue to explore the effects of perioperative glucocorticoids on blood pressure and blood glucose.

### Strengths and Limitations

Indeed, all our findings should be verified and extended in larger, preferably multi‐center studies that prospectively examine the influence of type, dose, and frequency of perioperative glucocorticoids with sufficiently long follow‐up to reliably detect differences in complications, including those that occur later, such as periprosthetic joint infection. In this way, the present work justifies closer consideration of perioperative glucocorticoids to alleviate pain in individuals with rheumatoid arthritis.

## Conclusion

Perioperative glucocorticoids may reduce the need for rescue opioids after total joint arthroplasty of rheumatoid arthritis patients, without increasing the incidence of acute complications, hypotension, or hyperglycemia.

## Conflict of Interest Statement

The authors have no relevant financial or non‐financial interests to disclose.

## Ethical Approval

Approval for the study protocol was obtained from the Medical Research Ethics Committee at Sichuan University and the local village committee, aligning with the principles outlined in the Declaration of Helsinki. Prior to their participation in the study, all patients provided informed consent, underscoring the commitment to ethical standards and the protection of participants' rights.

## Author Contributions

All authors had full access to the data of the study and take responsibility for the integrity and interpretation of the data. Conceptualization, YH Lai, ZY Luo, and ZK Zhou; methodology, YH Lai, C Huang; investigation, YH Lai, YR Cai; formal analysis, YH Lai, ZC Ding, and JX Fan; resources, ZK Zhou; writing—original draft, YH Lai; writing—review and editing YH Lai; visualization, ZY Luo; supervision, ZK Zhou.

## Funding Information

This work was supported by the National Key R&D Program of China (2022YFC2503104) and National Natural Science Foundation of China (82172394, U22A20280).

## Statement of Informed Consent

Informed consent was obtained from all individual participants included in the study.

## Statement of Human Animal Rights

The study was approved by the institution research ethics committee.
